# Supplementary material for: Preschool Teachers’ Psychological Distress and Work Engagement during COVID-19 Outbreak: The Protective Role of Mindfulness and Emotion Regulation
Source: Int J Environ Res Public Health. 2022 Feb 24;19(5):2645. doi: 10.3390/ijerph19052645 (PMC8909723; doi:10.3390/ijerph19052645)
Supplement: Supplementary file 1 [file ijerph-19-02645-s001.zip › ijerph-1542818-supplementary.pdf]

Call to Care: A program for teachers

Week	Mode	Objectives	Skills
1	Introduction	Getting to know the teachers<br>Introducing the program<br>Setting the rules for the training	Diaphragmatic breathing
2	Introduction	Introducing the theoretical framework<br>Teaching imagery of a "safe place"	"Safe place" imagery
3	Receiving care	Understanding the causes of burnout<br>Developing burnout prevention strategies<br>Teaching vagal breathing	Vagal breathing
4	Receiving care	Introducing the concept of mindfulness<br>Enhancing motivation to practice mindfulness	Brief mindfulness (5 min)
5	Receiving care	Introducing attachment theory<br>Deepening the process of mindfulness	Mindfulness (10 min)
6	Receiving care	Dealing with destructive feelings<br>Teaching compassion meditation	Mindfulness (15 min)<br>Compassion mediation – care moment
7	Receiving care	Dealing with barriers to receiving care<br>Deepening compassion meditation's practice	Mindfulness (15 min)<br>Compassion mediation – dear person
8	Receiving care	Designing experiences for receiving care<br>Teaching imagery of receiving care	Mindfulness (15 min)<br>Receiving care imagery
9	Self-care	Introducing the concept of self-compassion<br>Teaching self-compassion meditation	Meditation – self-compassion
10	Self-care	Evaluating level of self-care<br>Identifying needs for self-compassion<br>Designing daily self-compassion practices	Meditation – self-compassion

11	Self-care	Learning the differences between self-esteem and self-compassion<br>Practice self-affirmation	Meditation – self-compassion<br>Self-affirmation
12	Self-care	Developing awareness to their self-criticism<br>Learning ways to combat their self-criticism	Meditation – self-acceptance
13	Self-care	Identifying barriers for self-compassion<br>Practicing self-gratitude	Meditation – self-gratitude
14	Self-care	Introducing the concepts mind-sets<br>Develop growth mindset<br>Learning to flex rigid thoughts	Adopting growth mindset<br>Flexing rigid thoughts
15	Extending care	Exploring the benefits of giving to others<br>Practicing small care gestures toward others	Meditation – from receiving to giving
16	Extending care	Introducing the concepts of radical compassion<br>Learning to practice radical compassion<br>Learning empathic communication	Meditation – accepting challenging people<br>Empathic communication
17	Extending care	Learning the basis of human judgment<br>Teaching teachers perspective-taking	Meditation – accepting "difficult" students<br>Perspective taking
18	Extending care	Understanding stereotyping and prejudice<br>Teaching critical thinking	Critical thinking
19	Extending care	Introducing the bystander effect	Active bystandership
20	Extending care	Designing a students' project focusing on giving to the community	
